# Identification of long noncoding RNAs involved in resistance to downy mildew in Chinese cabbage

**DOI:** 10.1038/s41438-021-00479-1

**Published:** 2021-03-01

**Authors:** Bin Zhang, Tongbing Su, Peirong Li, Xiaoyun Xin, Yunyun Cao, Weihong Wang, Xiuyun Zhao, Deshuang Zhang, Yangjun Yu, Dayong Li, Shuancang Yu, Fenglan Zhang

**Affiliations:** 1 Beijing Vegetable Research Center (BVRC), Beijing Academy of Agriculture and Forestry Sciences (BAAFS), 100097 Beijing, China; 2 Key Laboratory of Biology and Genetic Improvement of Horticultural Crops (North China), Ministry of Agriculture, 100097 Beijing, China; 3 Beijing Key Laboratory of Vegetable Germplasm Improvement, 100097 Beijing, China

**Keywords:** Non-coding RNAs, Plant molecular biology

## Abstract

Brassica downy mildew, a severe disease caused by *Hyaloperonospora brassicae*, can cause enormous economic losses in Chinese cabbage (*Brassica rapa* L. ssp. *pekinensis*) production. Although some research has been reported recently concerning the underlying resistance to this disease, no studies have identified or characterized long noncoding RNAs involved in this defense response. In this study, using high-throughput RNA sequencing, we analyzed the disease-responding mRNAs and long noncoding RNAs in two resistant lines (T12–19 and 12–85) and one susceptible line (91–112). Clustering and Gene Ontology analysis of differentially expressed genes (DEGs) showed that more DEGs were involved in the defense response in the two resistant lines than in the susceptible line. Different expression patterns and proposed functions of differentially expressed long noncoding RNAs among T12–19, 12–85, and 91–112 indicated that each has a distinct disease response mechanism. There were significantly more *cis*- and *trans*-functional long noncoding RNAs in the resistant lines than in the susceptible line, and the genes regulated by these RNAs mostly participated in the disease defense response. Furthermore, we identified a candidate resistance-related long noncoding RNA, *MSTRG.19915*, which is a long noncoding natural antisense transcript of a *MAPK* gene, *BrMAPK15*. Via an agroinfiltration-mediated transient overexpression system and virus-induced gene silencing technology, *BrMAPK15* was indicated to have a greater ability to defend against pathogens. *MSTRG.19915*-silenced seedlings showed enhanced resistance to downy mildew, probably because of the upregulated expression of *BrMAPK15*. This research identified and characterized long noncoding RNAs involved in resistance to downy mildew, laying a foundation for future in-depth studies of disease resistance mechanisms in Chinese cabbage.

## Introduction

In recent years, with the development of advanced sequencing techniques, the characterization of the transcriptional landscape has been extensively explored, leading to an improved understanding of certain biological processes in plants^1–3^. Studies have shown that nearly 90% of the genome, including protein-coding mRNAs (only 2%) and noncoding RNAs (ncRNAs), which have little or no coding ability, is transcribed^4^. Long noncoding RNAs (lncRNAs) are kinds of ncRNAs with a length >200 nt^5^. LncRNAs are classified as long intergenic noncoding RNAs (lincRNAs), long noncoding natural antisense transcripts (lncNATs), and intronic RNAs (incRNAs) based on their genome location and as *cis*- or *trans*-acting lncRNAs based on their function^6,7^. Although lncRNAs have been identified recently in many plant species, information on the molecular mechanisms underlying lncRNAs is limited^8^. Studies have shown that lncRNAs could play roles in transcription, epigenetic, and posttranscriptional modification as signals, decoys, guides, or scaffolds^9^. *COLD Assisted Intronic noncoding RNA* (*COLDAIR*), an incRNA, was proven to silence the floral repressor *Flowering Locus C* (*FLC*) in Arabidopsis^10^. Another group of lncNATs functions as repressors of FLC, e.g., *COOLAIR* plays a crucial role in histone methylation at lysine 27 (H2K27me3) at the *FLC* locus^11^. Moreover, the lincRNA *ELF18-Induced Long Noncoding RNA 1* (*ELENA1*) can enhance resistance to *Pseudomonas syringae* by regulating the expression level of *pathogenesis-related 1* (*PR1*)^12^. LncRNAs that are present at the same chromosomal locus as their regulated genes are termed *cis*-functional lncRNAs; otherwise, they are termed *trans*-functional lncRNAs^7^. For example, the *cis*-functional lncRNA, *antisense Delay of Germination 1* (*as-DOG1*) functions as a repressor of seed dormancy^13^. Some *tr*ans-functional lncRNAs can bind microRNAs (miRNAs) to interrupt the interaction with their target mRNAs, the phenomenon of which is known as endogenous target mimicry (eTM)^7^. Two *trans*-functional lncRNAs, *slylnc0195* and *slylnc1077*, were found to act as target mimics of miR-166 and miR-399, respectively, and are involved in tomato yellow leaf curl virus (TYLCV) resistance^3^. Increasing numbers of lncRNAs have been identified to participate in various plant biological processes and are worthy of further exploration.

Chinese cabbage (*Brassica rapa* L. ssp. *pekinensis*), a kind of Brassica plant in the Cruciferae family, is one of the most important vegetable crop plants in China, with great economic value^14^. Brassica downy mildew is a fungal disease caused by infection with the oomycetes of *Hyaloperonospora parasitica* var. *Brassicae* and is one of the most severe diseases affecting Chinese cabbage production. To counteract various harmful pathogens in their natural environment, plants have evolved innate immune systems, of which there are many different defense-related gene families, such as mitogen-activated protein kinase (MAPK), nucleotide-binding site (NBS), receptor-like kinase (RLK) protein and receptor-like protein (RLP) families, and phytohormones, such as salicylic acid (SA), jasmonic acid (JA), ethylene (ET), abscisic acid (ABA), and brassinosteroids (BR), involved^15–19^. To date, some crucial downy mildew resistance genes and loci have been identified in Arabidopsis, the Cruciferae model plant species, and in *Brassica* plants. In Arabidopsis, *Powdery Mildew Resistant 4* (*PMR4*) and *Rab GTPase homolog 4c* (*RabA4c*) were found to play roles in callose deposition in downy mildew resistance, and some genes in the SA and JA signaling pathways are involved in the response to downy mildew, such as *PR*s, *NPR*s, and *WRKY*s^20,21^. With respect to *Brassica*, some resistant loci have been mapped in broccoli using molecular markers, and two single-nucleotide polymorphisms (SNPs) related to downy mildew resistance have also been identified in *Brassica napus*^22,23^. In Chinese cabbage, major resistance-related quantitative trait loci (QTLs) at different developmental stages have been identified via QTL mapping methods or genome-wide association study (GWASs), including the following: *BraDM* on A08; *BrRHP1* on A01; and six QTLs on A04, A06, and A08^24–27^. After downy mildew infection, the expression patterns of major genes in the SA and JA signaling pathways imply that SA is the main plant hormone involved in downy mildew resistance^28,29^. Although some candidate downy mildew resistance genes and loci have been identified, their molecular mechanisms are not fully characterized in Chinese cabbage.

In the signaling pathways of the plant defense response, the expression of functional genes can be transcriptionally and posttranscriptionally regulated^30^. Initially, the idea of lncRNAs as transcriptional or posttranscriptional regulators was neglected due to their low expression level and low sequence conservation. Recently, plant lncRNAs functioning in photomorphogenesis, organogenesis in roots, flowering time control, abiotic stress responses, and reproduction have been increasingly reported; however, until now, many fewer defense-related lncRNAs have been identified. In melon, there 611 lncRNAs were found to be differentially expressed after powdery mildew infection in a resistant melon line^31^. In Arabidopsis, *LINC*-*AP2*, a lincRNA, was found to negatively regulate the expression of the *AP2* gene during turnip crinkle virus infection^32^. *LncRNA16397*, an antisense transcript of *SIGRX22*, was found to enhance resistance to *Phytophthora infestans* by regulating the expression of *SIGRX*s in tomato^33^. Another immunity-related lncRNA in tomato, *lncRNA33732*, was proven to interact with the W-box element in *WRKY*1’s promoter to activate the induction of *RBOH* expression mediated by WRKY1, which is involved in H_2_O_2_ accumulation during *P. infestans* infection^34^. Moreover, lncRNA39026 was suggested to induce the expression of *PR* genes and decoy miR168a, which could enhance resistance to *P. infestans* in tomato^35^. In cotton, two lncNATs, *GhlncNAT-ANX2* and *GhlncNAT-RLP7*, were found to participate in resistance to *Verticillium dahlia* and *Botrytis cinerea*, possibly associated with the increased expression levels of *LOX1* and *LOX2*^36^. Although lncRNAs in nonheading Chinese cabbage and those expressed during pollen developmental stages in *B. rapa* have been identified, there have been no reports of immunity-related lncRNAs in *B. rapa*^37,38^.

In this study, we first characterized defense-related lncRNAs in response to downy mildew in two resistant (T12–19 and 12–85) and one susceptible (91–112) Chinese cabbage line. A total of 3711 lncRNAs were identified in response to downy mildew in Chinese cabbage. Different resistance responses, based on the expression profiles of both mRNA and lncRNAs, between the resistant and susceptible lines were demonstrated. Furthermore, we tried to explore candidate resistance-related lncRNAs involved in the defense response by regulating the expression of protein-coding genes. Our findings will shed light on the molecular mechanism underlying downy mildew resistance in Chinese cabbage.

## Results

### Gene expression profiles of downy mildew-resistant and downy mildew-susceptible lines of Chinese cabbage after downy mildew inoculation

**Fig. 1:**
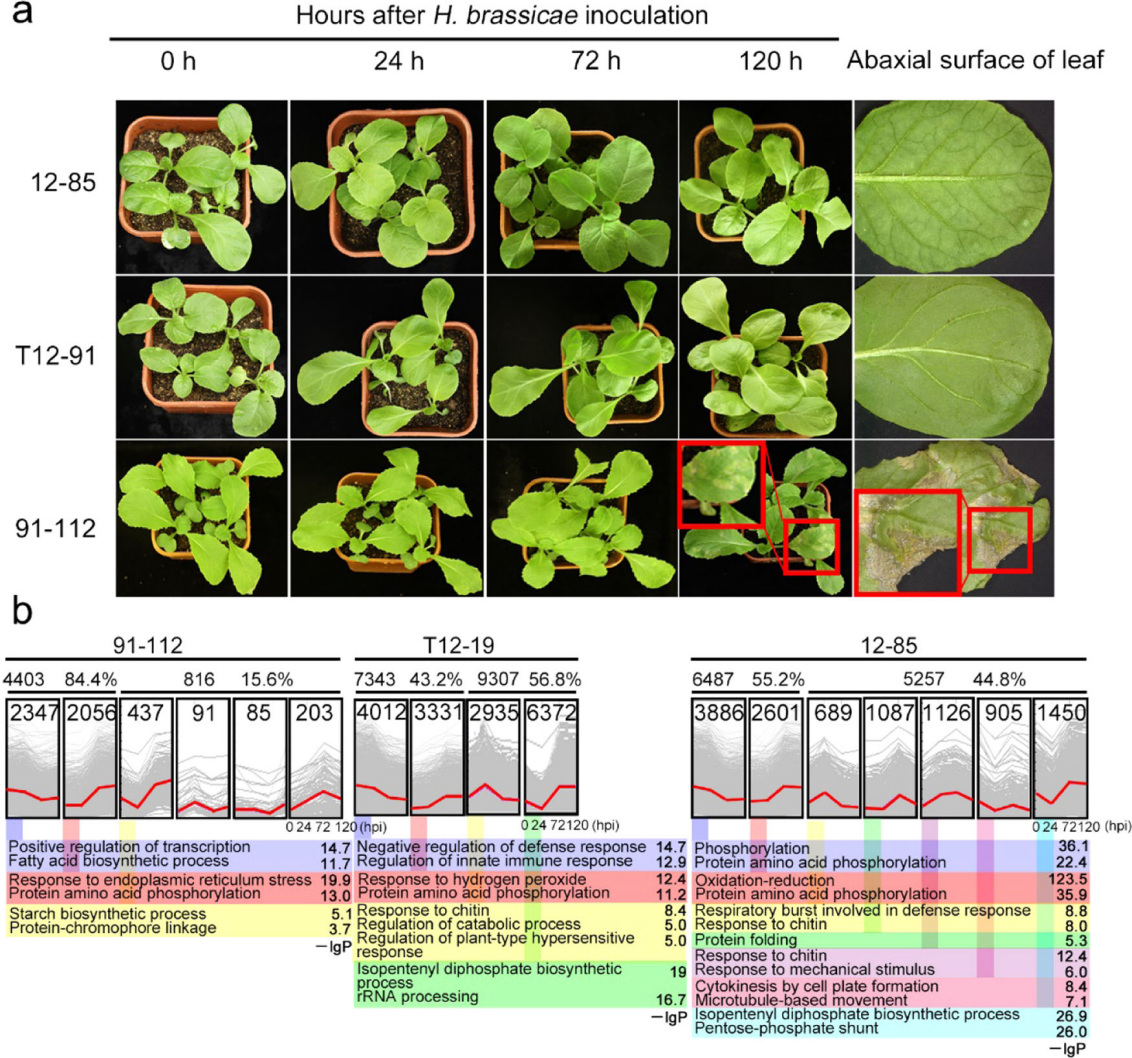
Disease incidence of 12–85, T12–19, and 91–112 seedlings and expression patterns of DEGs after infection with *H. brassicae*. **a** Incidence of infection of 12–85, T12–19, and 91–112 seedlings at 0, 24, 72, and 120 hpi. The susceptible line, 91–112, exhibited disease symptoms at 72 hpi, while there were no symptoms on any leaves of the two resistant lines, 12–85 and T12–19, at any time. The images in the small red squares show the overhead views of the seedlings in each panel. **b** Different expression patterns of DEGs in 91–112, T12–19, and 12–85. In each image of the cluster, the gray lines represent the normalized expression values of the genes, and the red line indicates the centroid graph, which is considered to be the expression trend of these genes within a single cluster. The gene numbers are at the top of each cluster image. Based on the –lg*P* value (shown behind the GO terms), the top two or three GO items are highlighted under the clusters

To investigate the gene expression profiles in response to Brassica downy mildew, transcriptome sequencing of disease-resistant (12–85 and T12–19) and disease-susceptible lines (91–112), including the quantification of mRNA and lncRNAs, after pathogen invasion was performed. Three-week-old seedlings were inoculated with *H. brassicae*. The leaves of the 12–85, T12–19, and 91–112 seedlings showed no obvious changes at 24 h post inoculation (hpi) compared with those at 0 hpi. At 72 and 120 hpi, most of the leaves of 91–112, but not those of 12–85 or T12–19, turned yellow due to the high-humidity conditions required for pathogen invasion (Fig. 1a). White spores and spore stems of *H. brassicae* began to appear at 72 hpi on the abaxial surface of leaves of susceptible line 91–112. At 120 hpi, the leaves of 91–112 displayed obvious disease symptoms, while no changes were observed in resistant lines 12–85 and T12–19 (Fig. 1a). Thus, downy mildew inoculation was effective, and leaf samples were collected at 0, 24, 72, and 120 hpi.

Thirty-six samples (three biological repeats each) were collected for transcriptome sequencing and analysis. We obtained more than 80 million clean reads from each sample, 60–70% of which were mapped onto the *B. rapa* reference genome (v2.5) (Fig. S1a). Approximately 50–60% of the clean reads in each sample were mapped to a unique gene (Fig. S1b). Detailed information on the sequenced samples is provided in Table S1. Through Pearson correlation analysis of these samples based on the fragments per kilobase million (FPKM) values of all the transcripts, a strong correlation occurred among three biological repeats (Table S2). Principal component analysis (PCA) showed that the 0 h samples (the white shapes in the associated figure) clustered more closely together with the 24 h samples (the yellow shapes) than with the 72 and 120 h samples (the blue and green shapes), which indicated that the gene expression at 72 and 120 hpi was more responsive to pathogen invasion than that at 24 hpi (Fig. S1c). The DEGs were obtained from the comparisons between samples at 24, 72 and 120 hpi and those at 0 hpi (listed in Table S3). At each time point, the resistant lines 12–85 and T12–19 had more responsive genes (whose expression was either upregulated and downregulated) than the susceptible line 91–112 did (Fig. S2a, b). A Venn diagram showing the number of DEGs at 24, 72, and 120 hpi was constructed; the diagram details the common and exclusive DEGs among 91–112, 12–85, and T12–19 (Fig. S2c). The GO terms (with the top 20 –lg*P* values) of exclusive DEGs whose expression was upregulated and downregulated revealed that there were more defense-related terms (highlighted in yellow) in the resistant lines than in the susceptible line (Fig. S2d, e). The common DEGs among 91–112, 12–85, and T12–19 were mainly enriched in photosynthesis, metabolic process, and response to stimulus terms (Fig. S3a). The common DEGs found only in the resistant lines, 12–85 and T12–19, were enriched in immune system processes, including the response to chitin and respiratory burst, which are involved in the defense response (Fig. S3b). All the –lg*P* values of these GO terms are shown in Table S4. In summary, the gene number and GO analysis of the DEGs demonstrated that there were more DEGs involved in the disease defense response in the two resistant lines than in the susceptible line, and the resistance mechanisms of the two resistant lines were obviously different.

We clustered all these DEGs according to their expression patterns during 0–120 hpi in 91–112, 12–85, and T12–19 (Fig. 1b). The results showed that the expression of most of DEGs was continuously upregulated or downregulated, and only 15.6% of the DEGs were responsive to the pathogen at a certain time point in 91–112, whereas 44.8% and 56.8% were responsive in 12–85 and T12–19, respectively (Fig. 1b). The top two or three most significantly enriched GO terms of each cluster showed that the DEGs whose expression was continuously upregulated or downregulated were mostly enriched in protein amino acid phosphorylation in 91–112, 12–85, and T12–19. In addition, oxidation–reduction was enriched in the DEGs whose expression was continuously upregulated in 12–85; the GO terms associated with processes involving the negative regulation of the defense response and the regulation of the innate immune response were enriched in the DEGs whose expression was continuously downregulated in T12–19 (Fig. 1b). For the stage-specific high-expression or low-expression DEGs, several defense-related and immunity-related GO items were enriched in the resistant lines but not in susceptible line 91–112. In 12–85, the 24 hpi-specific high-expression DEGs were enriched in the respiratory burst involved in the defense response and response to chitin, and the 24 and 72 hpi-specific high-expression DEGs were enriched in the response to chitin and the response to mechanical stimuli (Fig. 1b). In T12–19, the 24 hpi-specific high-expression DE genes were enriched in the response to chitin, regulation of catabolic processes, and regulation of plant-type hypersensitive responses (Fig. 1b).

### Identification and characterization of lncRNAs in Chinese cabbage

**Fig. 2:**
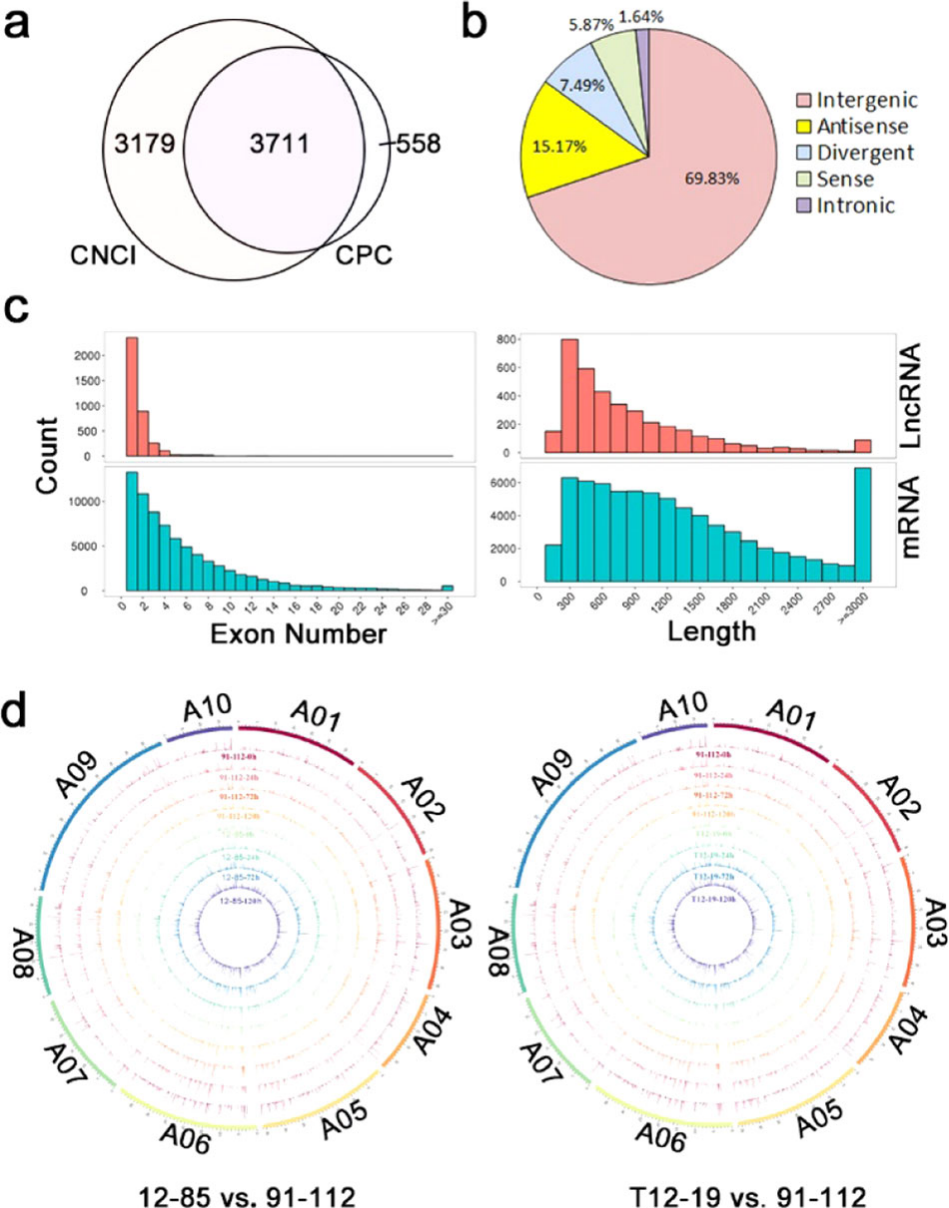
Identification and characterization of lncRNAs in 91–112, 12–85, and T12–19. **a** Venn diagram analysis of identified lncRNAs using the CNCI and CPC software. **b** Proportions of different kinds of lncRNAs. **c** Comparison of the exon number and transcript length between lncRNAs and mRNAs. **d** Expression levels and genomic locations of all the lncRNAs in 91–112, 12–85, and T12–19. 12–85 compared with 91–112 is shown on the left, and T12–19 compared with 91–112 is shown on the right

From more than 80 million clean reads in each sample, we identified lncRNAs according to their read coverage, transcript length and protein coding potential. A total of 3711 lncRNAs were ultimately identified in the sequenced samples (Fig. 2a). The associated results from StringTie for the predicted lncRNAs are shown in Table S5. The identified lncRNAs were divided into lincRNAs, incRNAs, lncNATs, sense lncRNAs and divergent lncRNAs (uncertain type) based on their genomic location. The percentages of different types of lncRNAs are shown in Fig. 2b. The most abundant class was lincRNAs (69.83%), followed by lncNATs (15.17%), and the least abundant were incRNAs (Fig. 2b). All the identified lncRNAs and their classified types are shown in Table S6. The exon number and length of the lncRNAs were significantly lower than those of mRNAs (Fig. 2c). Few lncRNAs contained more than four exons; however, some lncRNAs contained more than 30 exons (Fig. 2c). LncRNAs with a length similar to that of mRNAs accounted for less than one-tenth of the mRNAs (Fig. 2c). By the use of the Circos program^39^, an expression distribution of lncRNAs from 12–85, T12–19, and 91–112 along 10 *B. rapa* chromosomes (A01–A10) was constructed (Fig. 2d). These lncRNAs exhibited no obvious preference for any particular genomic location (Fig. 2d). The expression levels of all the identified lncRNAs are listed in Table S7. Among the 3711 lncRNAs, the genomic loci of 2591 intergenic, 61 intronic, 218 sense, 563 antisense, and 278 divergent lncRNAs could be distinguished (Fig. S4).

### Pathogen-induced expression profiles of lncRNAs in resistant and susceptible lines

**Fig. 3:**
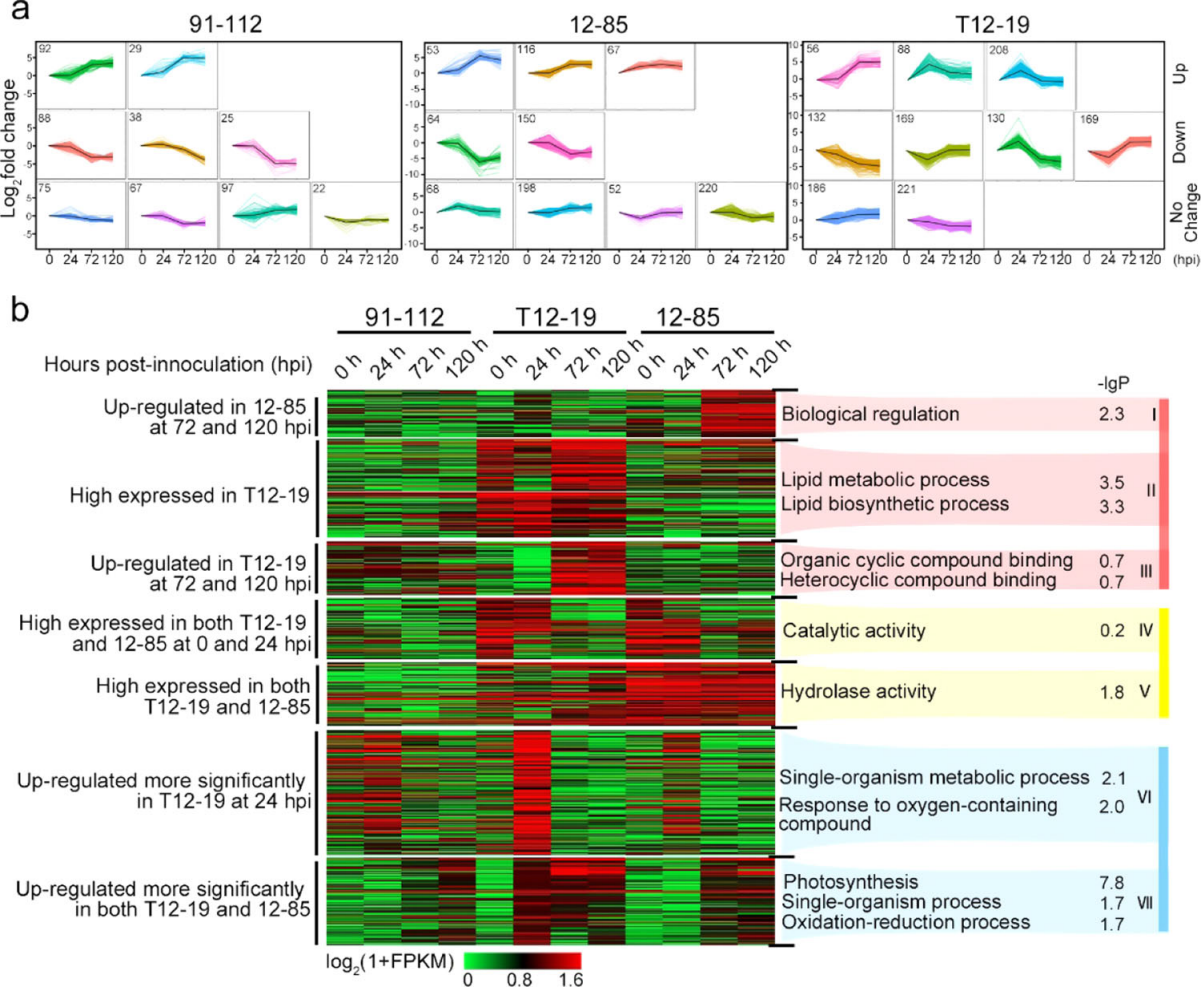
Characterization of the expression regulation of lncRNAs after *H. brassicae* infection. **a** Expression patterns of lncRNAs in 91–112, 12–85, and T12–19. The numbers of lncRNAs in each cluster are marked in the top left corner. All the clusters were classified into “up”, “down”, and “no change” groups. **b** Clustering analysis of differentially expressed lncRNAs. Seven clusters were identified based on the different induction patterns in 91–112, T12–19, and 12–85. The expression levels of lncRNAs in the first three clusters (highlighted in red) were upregulated specifically in one resistant line. In clusters IV and V (highlighted in yellow), the lncRNAs exhibited a similar induction pattern in the two resistant lines, but the pattern differed from that of the susceptible line. The lncRNAs in the last two clusters (highlighted in blue) exhibited similar expression patterns in all three lines; a greater induction intensity was observed in the resistant lines than in the susceptible line. The top three significant GO items enriched by the regulated genes of lncRNAs in each cluster are shown on the right. The –lg*P* values were shown behind the GO terms

To characterize the expression of lncRNAs in response to pathogen invasion, the induction patterns and numbers of lncRNAs were investigated. The expression of lncRNAs at 24, 72, and 120 hpi was compared with that at 0 hpi. LncRNAs with a FPKM value of less than one in both compared samples were not used for this comparison. The numbers of lncRNAs whose expression was upregulated or downregulated among the four types showed no significant differences between the different samples (Fig. S5). The lncRNAs whose expression was upregulated gradually increased from 24 to 120 hpi in all three inbred lines, while markedly more increased lncRNAs, especially lncNATs, from 24 to 72 hpi were observed in T12–19 compared with 91–112 (Fig. S5). This suggested that the lncRNAs in the resistant line T12–19 might have a more pronounced response in the defense process. The expression levels at 0 hpi were designated as controls, and the log_2_(fold change) values were applied for clustering analysis. On the basis of the pattern of changing values, nine groups clustered together using the *K*-means method in 91–112, 12–85, and T12–19 (Fig. 3a). The lncRNAs with average log2 values of more than −2 and <2 were considered to belong to the “no change” group, whereas those with average values >2 and <−2 were divided into the upregulated and downregulated groups, respectively (Fig. 3a). The results showed that approximately half of the lncRNAs were in the “no change” group in 91–112 and 12–85 but only 30.6% for T12–19 (Fig. 3a). The expression of a higher proportion of lncRNAs was obviously upregulated and downregulated in T12–19, including a cluster of 130 lncRNAs (green) whose expression was upregulated at 24 hpi and downregulated at 72 and 120 hpi and a cluster of 169 lncRNAs (red) whose expression was downregulated at 24 hpi and upregulated at 72 and 120 hpi (Fig. 3a).

To clarify in which signaling pathways the lncRNAs participate in response to pathogen infection, we clustered the differentially expressed lncRNAs based on their expression levels in all the samples, and each cluster was subsequently subjected to GO analysis. The clusters with similar expression patterns between the susceptible and resistant lines and those with irregular patterns were not used for GO analysis. In cluster I, lncRNAs were highly expressed in 12–85 and found to be involved in biological regulation (Fig. 3b). The lncRNAs whose expression was specifically upregulated in T12–19 at 0–120 hpi (cluster II) were mostly involved in lipid biosynthesis and metabolic processes (Fig. 3b). The expression of the lncRNAs of cluster III was specifically upregulated in T12–19 at 72 and 120 hpi, and these lncRNAs were found to be involved in organic cyclic and heterocyclic compound binding (Fig. 3b). The lncRNAs in the remaining clusters (clusters IV, V, VI, and VIII) exhibited similar expression patterns in the two resistant lines and were found to be mainly enriched in oxidation–reduction processes, responses to oxygen-containing compounds, photosynthesis, single-organism process hydrolase activity, and catalytic activity (Fig. 3b). In addition, in clusters VI and VII, the lncRNAs in T12–19 were more responsive to pathogen invasion than were those in 12–85 (Fig. 3b). Briefly, there were more responsive lncRNAs in the two resistant lines than in the susceptible line 91–112. Furthermore, the responsive lncRNAs might function mainly in lipid metabolic processes and oxidation reactions to provide plant disease resistance.

### Characterization of specific *cis*- and *trans*-functional lncRNAs in resistant and susceptible lines

We identified the *cis*- and *trans*-functional lncRNAs and their target genes, which are listed in Table S6. We first investigated the number of differentially expressed lncRNAs at each time point compared with 0 hpi. There were more differentially expressed *cis*-functional lncRNAs than *trans*-functional lncRNAs (Fig. S6a). In addition, there were significantly more differentially expressed lncRNAs, both *cis*- and *trans*-functional ones, in the resistant lines T12–19 and 12–85 than in the susceptible line 91–112 (Fig. S6a). At 24 hpi, the difference in the number of differentially expressed lncRNAs among these three inbred lines was the greatest. There were almost no differentially expressed lncRNAs in 91–112, and the number of differentially expressed lncRNAs in T12–19 was almost three times greater than that in 12–85 (Fig. S6a). These results showed that, compared with the susceptible line, the resistant lines were more responsive to pathogen invasion, especially at 24 hpi, and compared with regulation by *trans*-functional lncRNAs, regulation by *cis*-functional lncRNAs was more common in disease resistance. Venn diagram analysis of differentially expressed lncRNAs in the two resistant lines and susceptible line at each time point was subsequently performed (Fig. S6b). The specific *cis*- and *trans*-functional lncRNAs in the resistant and susceptible lines were then defined.

**Fig. 4:**
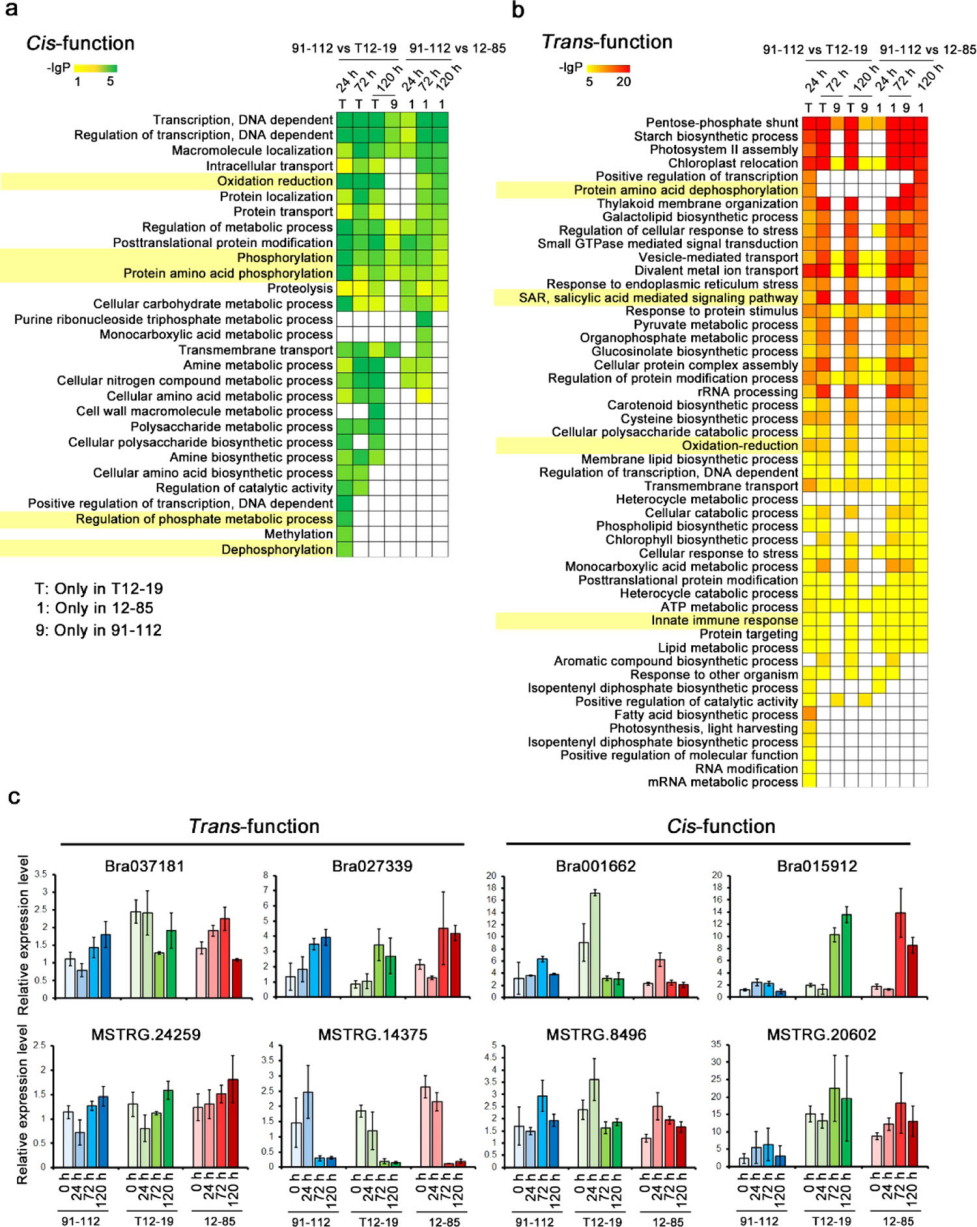
Functional analysis of *trans*- and *cis*-functional lncRNAs/protein-coding gene pairs. GO analysis of *cis*- (**a**) and *trans*-functional lncRNAs (**b**) at each time point in 91–112, 12–85, and T12–19. The top 20 significant GO items at each time point are shown. Several putative disease resistance response-related GO items are highlighted in yellow. **c** Relative expression levels of two pairs of *trans*-function lncRNAs/protein-coding genes and *cis*-function lncRNAs/protein-coding genes in all samples. One biological repeat of the 91–112 0 h sample was designed as 1. The values represent the means ± SDs (*n* = 3) of three biological replicates

A lncRNA and its regulated protein-coding gene form a lncRNA/protein-coding gene regulatory pair. The function of the protein-coding gene can be defined as the function of this regulatory pair. Based on KEGG pathway and GO analysis, the lncRNA/protein-coding gene regulatory pairs involved in the disease response were identified and are listed in Table S8. In addition, we also characterized the functions of *cis*- and *trans*-functional lncRNAs by GO enrichment analysis of their target genes. Many GO terms related to phosphorylation were enriched in *cis*-functional lncRNA-regulated genes in both T12–19 and 12–85. Interestingly, cellular compound biosynthesis- and metabolic process-related GO terms were enriched specifically in T12–19 (Fig. 4a). Compared with the *cis*-functional lncRNAs, the *trans*-functional lncRNA-regulated genes were enriched in some GO terms directly involved in the plant immune response, including SA-mediated signaling pathways and the innate immune response, in addition to those related to phosphorylation, in both T12–19 and 12–85 (Fig. 4b). There were only a few GO terms that were enriched in both *cis*- and *trans*-functional lncRNA-regulated genes in 91–112 because of the smaller number of differentially expressed lncRNA/protein-coding gene pairs in that line compared with the other lines. All the –lg*P* values of these GO terms are shown in Table S4. These results indicated that protein phosphorylation-related genes, which might be involved in signaling pathways leading to disease resistance, were regulated by both *cis*- and *trans*-functional lncRNAs. As such, the resistance mechanism in T12–19 might be significantly different from that in 12–85. The *cis*-functional lncRNA-regulated genes, which participate in cellular compound metabolic processes, were specifically involved in the resistance response in T12–19.

Among the regulated genes of all the *trans*- and *cis*-functional lncRNAs, some were found to be involved in the disease resistance response. From these regulatory pairs, the lncNAT or *trans*-functional lncRNAs coexpressed together with their target genes were selected as candidate lncRNA/protein-coding gene pairs. We performed quantitative real-time reverse transcription polymerase chain reaction (qRT-PCR) to verify their expression patterns during pathogen invasion (Fig. 4c). The Ct values from qRT-PCR are listed in Table S9. The expression patterns were consistent with those in the RNA-seq data, which are shown in Fig. S7, as calculated by their FPKM values. *MSTRG.24259*/*Bra037181* and *MSTRG.14375*/*Bra027339* are two pairs of *trans*-functional lncRNA/protein-coding gene pairs. The expression patterns of *MSTRG.24259* were similar to those of *Bra037181*, while the expression patterns of *MSTRG.14375* were the opposite of those of *Bra027339* (Fig. 4c). *Bra037181* is the homolog of *At5g66210*, which encodes calcium-dependent protein kinase 28 (CPK28), which was found to be involved in the plant defense response^40^. *Bra027339* encodes a peroxisomal enzyme, glycolate oxidase (GOX), and is homologous to *At3g14415* (*GOX2*) in Arabidopsis, which has been proven to participate in nonhost resistance^41^. For two *cis*-functional lncRNA/protein-coding gene pairs, *Bra001662*/*MSTRG.8496* and *Bra015912*/*MSTRG.20602*, the lncRNAs and protein-coding genes exhibited highly similar expression patterns during pathogen infection (Fig. 4c). *Bra001662* encodes a protein that is annotated as pto-interacting protein 1-like, which was identified as negatively regulating immune signaling in rice^42^. The encoded protein of *Bra015912* was annotated as MYB-related protein 308-like, which has putative functions in plant–pathogen interactions according to the KEGG pathway analysis. In conclusion, through GO-enrichment analysis and detection of the expression of lncRNA/protein-coding gene pairs, most lncRNAs appeared to contribute to the disease resistance response by regulating the expression of resistance-related genes during pathogen invasion.

### Candidate functional lncRNAs in resistance to Brassica downy mildew

**Fig. 5:**
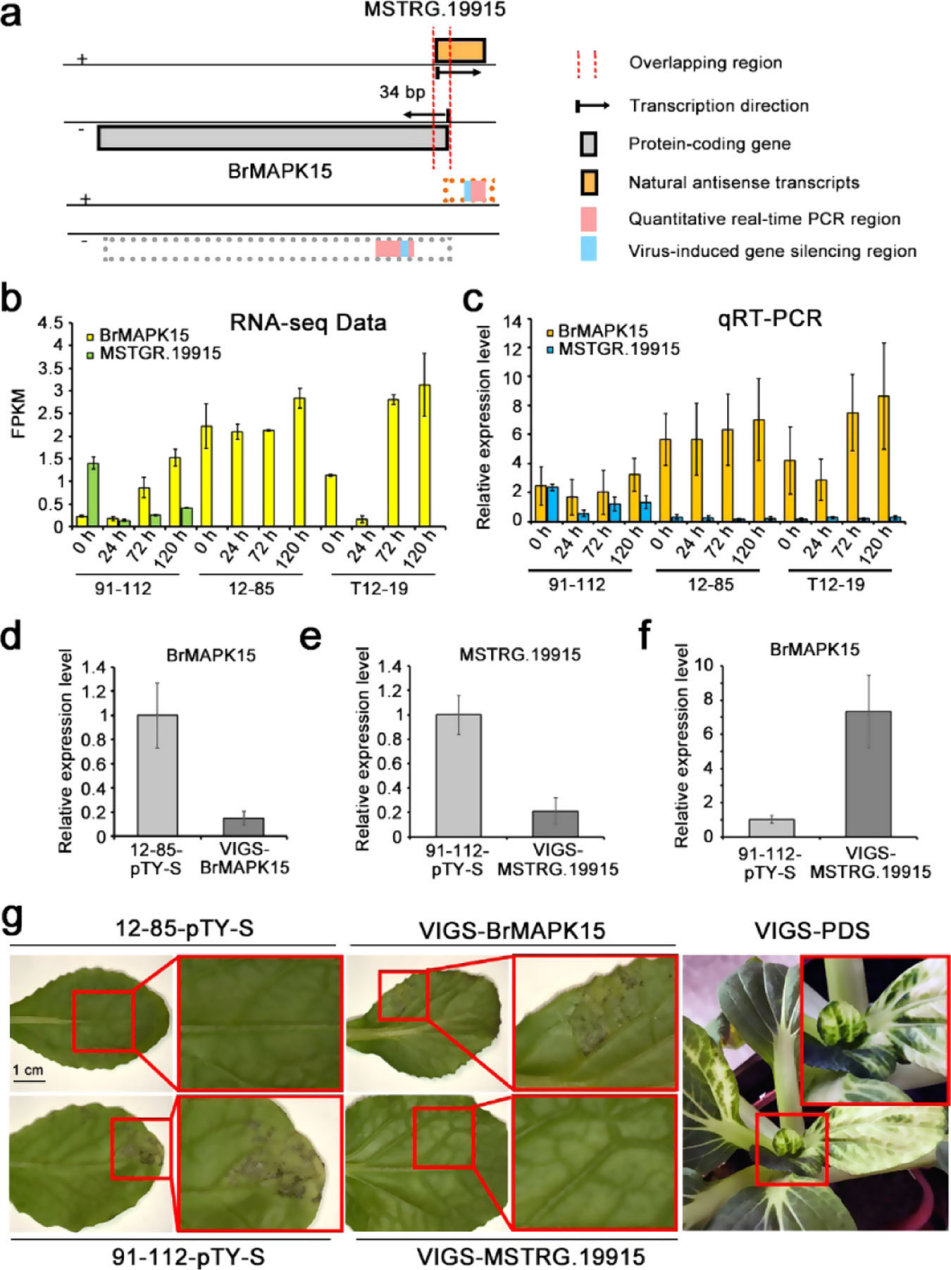
Functional verification of *MSTRG.19915* and *BrMAPK15* in downy mildew resistance using VIGS. **a** Schematic diagram of the genomic location, transcription direction, and locations of designed primers for *MSTRG.19915* and *BrMAPK15*. Specific primers (avoiding overlapping and conserved regions) were designed to ensure the silencing specificity and reliability for each target. **b** FPKM values of *MSTRG.19915* and *BrMAPK15* in the sequenced samples. **c** Relative expression levels of *MSTRG.19915* and *BrMAPK15* in sequenced samples according to qRT-PCR. **d** Change in the expression level of *BrMAPK15* after silencing *BrMAPK15* in 12–85; **e** Change in the expression level of *MSTRG.19915* after silencing *MSTRG.19915* in 91–112; **f** Change in the expression level of *BrMAPK15* after silencing *MSTRG.19915* in 91–112; **g** The phenotypes of 12–85, the *BrMAPK15*-silenced line, 91–112 and the *MSTRG.19915*-silenced line were investigated 5 days after *H. brassicae* inoculation. The PDS silencing line was used as a positive control for the VIGS method. The values represent the means ± SDs (*n* = 3) of three biological replicates

LncNATs are located in the coding region of a protein-coding gene in the reverse transcription direction. They are assumed to function as complementary nucleotides to suppress the expression of the target gene. From our data, we identified 521 lncNATs, among which 118 were expressed in response to pathogen invasion. We then detected the expression of the target genes of these differentially expressed lncNATs. The lncNATs that were coexpressed together with their target genes during *H. brassicae* infection were considered to be candidate functional lncRNAs in resistance to Brassica downy mildew. We found a lncNAT, *MSTRG.19915*, that targets a MAPK gene designated *BrMAPK15* due to its closest relationship with AtMAPK15 (Fig. S8). Their overlapping region was located within the first 36 bp of *BrMAPK15*, which probably reduces the transcriptional efficiency of *BrMAPK15* (Fig. 5a). According to the RNA-seq data, *MSTRG.19915* was not expressed in the two resistant lines, whereas the expression of *BrMAPK15* was much higher in the resistant lines than in the susceptible line (Fig. 5b). qRT-PCR was performed to verify the reliability of the expression results according to the RNA-seq data. The qRT-PCR expression patterns were consistent with those of the RNA-seq data (Fig. 5c). Therefore, *MSTRG.19915*, which is coexpressed together with its target gene *BrMAPK15*, was identified as a candidate resistance-related lncNAT.

To investigate the functions of *MSTRG.19915* and *BrMAPK15* in resistance to *H. brassicae*, we attempted to obtain loss-of-function lines using virus-induced gene silencing (VIGS) technology. We inserted the target sequences of *MSTRG.19915* and *BrMAPK15* into a pTY-S vector, which reconstituted turnip yellow mosaic virus (TYMV). The locations of the target sequences are shown in Fig. 5a. A high quantity of high-quality recombinant pTY-S plasmids were applied for inoculation. *BrMAPK15* was knocked down in resistant line 12–85, and *MSTRG.19915* was silenced in susceptible line 91–112. Seedlings infiltrated with empty PTY-S vectors were used as negative controls. Silencing of the *phytoene desaturase* (*PDS*) gene, which can cause white leaves to appear, was used as a positive control for the VIGS experiment. Gene silencing was detected at 2 weeks post-infiltration. Specific primers for qRT-PCR were designed to avoid the overlapping region (marked in Fig. 5a). The Ct values from qRT-PCR are listed in Table S9. The expression levels of *MSTRG.19915* and *BrMAPK15* were nearly five times lower in the 91–112 and 12–85 silenced lines, respectively, than in the control line (Fig. 5d, e). *MSTRG.19915* had a 36-bp region overlapping with that of *BrMAPK15* at the transcription initiation site. The expression level of *BrMAPK15* was probably altered by *MSTRG.19915* silencing. Therefore, we measured the expression of *BrMAPK15* in *MSTRG.19915*-silenced seedlings. The results showed that the expression of *BrMAPK15* increased by approximately sevenfold in the *MSTRG.19915*-silenced line, which indicated that *MSTRG.19915* could directly regulate the expression of *BrMAPK15* (Fig. 5f). After confirmation of the silencing of *MSTRG.19915* and *BrMAPK15*, the seedlings were inoculated with *H. brassicae*. Five days after pathogen invasion, the *BrMAPK15*-silenced seedlings presented disease symptoms on their leaves (5 susceptible among 30 seedlings), whereas the 12–85 seedlings transfected with empty pTY-S and inoculated with the pathogen were completely resistant to downy mildew (30 resistant seedlings among 30 total seedlings) (Fig. 5g). In the susceptible line 91–112, the susceptibility of the pTY-S-silenced seedlings was similar to that of 91–112 (27 susceptible seedlings among 30 total seedlings); however, 10 of the 30 seedlings exhibited the resistant phenotype after *MSTRG.19915* knockdown (Fig. 5g). Thus, silencing *BrMAPK15* could significantly reduce the disease resistance of 12–85, and silencing *MSTGR.19915* obviously enhanced the pathogen defense reaction of 91–112 (Fig. 5g). At ~3 weeks post-infiltration, the PDS-silenced seedlings showed the gradual appearance of white leaves (6 in 30 seedlings), which indicated that the VIGS system used in this study functioned correctly (Fig. 5g).

**Fig. 6:**
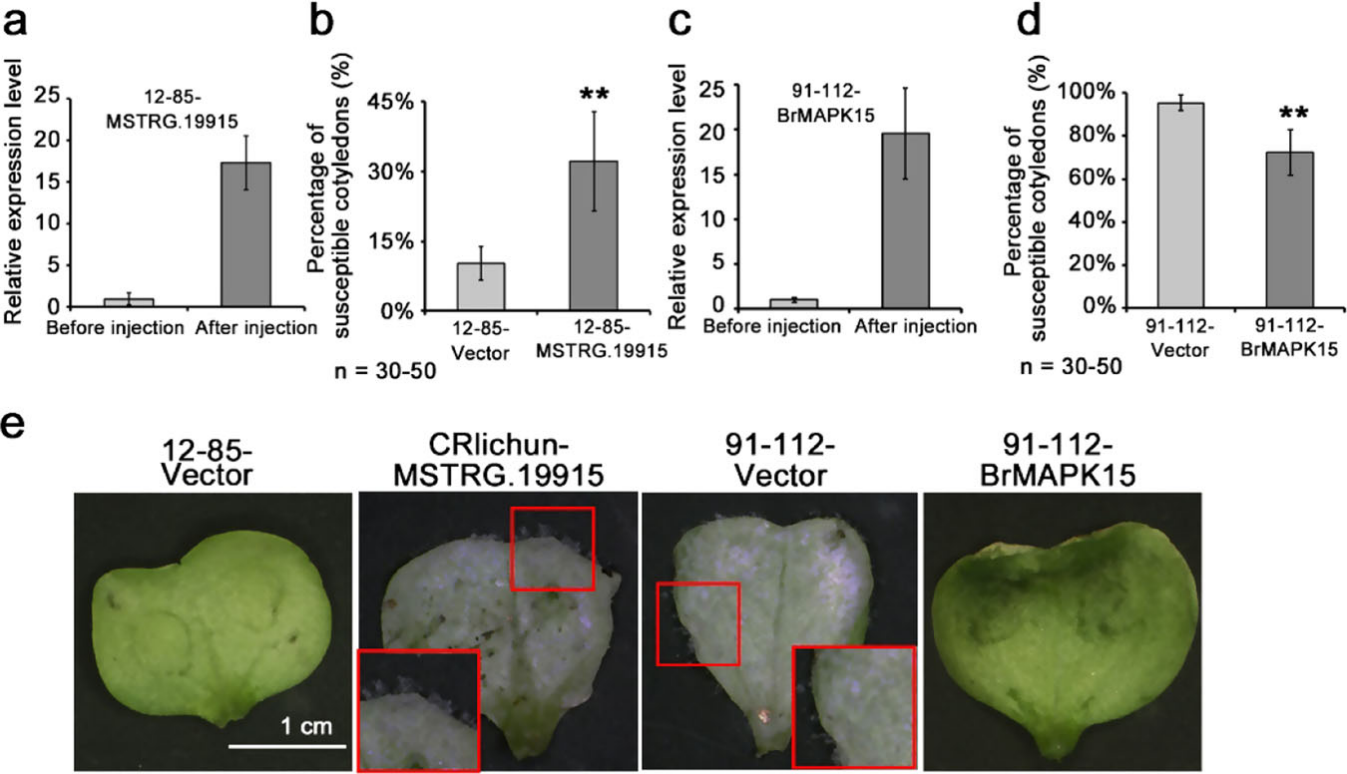
Functional analysis of *MSTRG.19915* and *BrMAPK15* in downy mildew resistance by agroinfiltration-mediated transient expression. Relative expression levels of *MSTRG.19915* (**a**) and *BrMAPK15* (**c**) overexpressed in 12–85 and 91–112 cells, respectively, were measured. The values represent the means ± SDs (*n* = 3) of three biological replicates. The expression of each gene before infiltration was defined as 1.0. The percentages of susceptible infiltrated cotyledons in *MSTRG.19915* overexpression lines (**b**) and *BrMAPK15* lines (**d**) after inoculation were investigated. Cotyledons of 12–85 and 91–112 infiltrated with empty vectors were used as the controls. The values represent the means ± SDs of three independent experiments. Each experiment involved 30–50 cotyledons. **e** The phenotypes of cotyledons infiltrated with *MSTRG.19915* and *BrMAPK15* were observed at 3 days after agroinfiltration. Leaves infiltrated with empty vectors were used as negative controls. The images in small red squares are magnified. The values represent the means ± SDs (*n* = 3) of three biological replicates

To further confirm the function of candidate lncRNA/protein-coding gene pairs in disease resistance, we tried to obtain overexpression lines. The construction of transgenic seedlings was time consuming and inefficient. However, the agroinfiltration-mediated transient expression system was successfully applied to investigate the functions of candidate genes in resistance to downy mildew in the cotyledons of *B. rapa*^43^. *MSTRG.19915* and *BrMAPK15* were inserted into pCAMBIA2300 vectors, and their overexpression was driven by the 35S promoter. Through the agroinfiltration-mediated transient expression system, the expression of *MSTRG.19915* increased by more than 17 times in 12–85 (Fig. 6a). The Ct values for gene quantification are listed in Table S9. Five days after inoculation, the disease symptoms of the infiltrated cotyledons were evaluated. The cotyledons infiltrated with empty vector were used as a control. Nearly 90% of the cotyledons (10.3% of susceptible cotyledons) infiltrated with the empty vector were resistant to downy mildew in 12–85 (resistant line) (Fig. 6b). However, 32.1% of 12–85 cotyledons were found to be susceptible after *MSTRG.19915* overexpression (Fig. 6b). Overexpression of *MSTRG.19915* enhanced the susceptibility of 12–85 (the amount of susceptible cotyledons increased by ~20%) (Fig. 6b, e). After infiltration, *BrMAPK15* was overexpressed nearly 20-fold in 91–112 (Fig. 6c). More than 90% (95.3%) of the 91–112 cotyledons exhibited susceptibility symptoms after empty vector infiltration (Fig. 6d). In contrast, only 72.4% of cotyledons of 91–112 appeared to be susceptible after overexpression of *BrMAPK15* (Fig. 6d). *BrMAPK15* overexpression in 91–112 could obviously significantly reduce susceptibility to downy mildew (Fig. 6d, e). Thus, the results showed that the lncNAT *MSTRG.19915* may be involved in the resistance response to *H. brassicae* by regulating the expression of a *MAPK* family gene, *BrMAPK15*, in *B. rapa*.

## Discussion

The downy mildew resistance mechanism of Chinese cabbage remains poorly understood, although recent research has made some progress^27,43^. Whole-genome-wide gene expression profiles may help gain insight into the disease resistance mechanism. A study on the expression profile of mRNAs in response to downy mildew in Chinese cabbage has been reported^2^. However, only immunity-related genes were listed, and neither GO-enrichment analysis nor cluster analysis was carried out in that article. For improved understanding of the resistance response to downy mildew at the transcriptional level, we assessed the transcriptional landscape of one susceptible (91–112) and two resistant lines (T12–19 and 12–85) using high-throughput RNA sequencing. Different Chinese cabbage cultivars usually exhibit distinct disease susceptibility to downy mildew. Therefore, we used two resistant lines, T12–19 and 12–85, with similar DIs to investigate the disease resistance mechanism. Intriguingly, T12–19 and 12–85 have distinctly different signaling pathways that respond to downy mildew. Among the genes whose expression was upregulated, plant hormone-related GO terms, such as the SA biosynthesis process and response to auxin, were more significantly enriched more in 12–85 than in T12–19. The GO terms involved in the cellular and organic substance metabolic process, such as the maltose metabolic process and thylakoid membrane organization, were specifically enriched in T12–19 (Fig. S2). These findings indicated that hormone signaling pathways might play a more important role in 12–85 than in the other lines during pathogen invasion. In T12–19, the genes enriched in metabolic processes of the biological macromolecular and cellular component categories were found to be involved in the disease response, which might imply that changes in the cellular structure, nutrients, energy levels, and redox status occurred during fungal infection.

We also identified lncRNAs and characterized the expression profiles of lncRNAs during *H. brassicae* invasion. A total 3711 lncRNAs were identified. Previously, 10,001 and 12,051 putative lncRNAs were identified under cold and heat treatment, respectively, in nonheading Chinese cabbage and after pollination in *B. rapa*^37,38^. The variation in lncRNA quantity was probably caused by different treatments, plant materials, identification methods, and genome versions used for mapping. Although the numbers of differentially expressed lncRNAs between resistant and susceptible lines were not significantly different, a larger number of *cis*- and *trans*-functional lncRNAs were detected in the two resistant lines than in the susceptible line (Figs. S5 and S6). This might demonstrate that the expression of lncRNAs coexpressed together with protein-coding genes increased after inoculation; however, the functions of most lncRNAs are unknown. The identified *cis*- and *trans*-functional lncRNAs might also play important roles in the response to disease resistance (Fig. 4a). From these data, lncRNA/protein-coding gene regulatory pairs involved in downy mildew defense in Chinese cabbage were obtained, which are listed in Table S8. Among these lncRNA-regulated protein-coding genes, some are considered to function in plant and pathogen interactions and in the plant immune system. Indeed, some of them are annotated as putative RLPs, which probably function as R proteins (Table S8). Members of the WRKY transcription factor family, which are involved in the defense response, were also included. These results provide the basis to clarify the function of lncRNAs that participate in downy mildew resistance.

It is quite difficult to verify the function of lncRNAs to understand RNA-mediated gene regulation^44^. To date, few lncRNAs have been functionally characterized, despite thousands of lncRNAs having been identified in many plant species^44^. In this study, we identified a candidate resistance-associated lncRNA, *MSTRG.1991*5, which is a lncNAT of *Bra003834*, a putative homolog gene of *AtMAPK15* based on the phylogenetic tree in Fig. S8. MAPK cascades are highly conserved signaling pathways that are involved in the regulation of plant development and are involved in a variety of stress responses, including those in response to pathogen infection, wounding, temperature, drought, and reactive oxygen species^18^. In the defense response, among that of 20 *AtMAPK* genes, the expression of *AtMAP3*, *AtMAP6*, *AtMAP4*, and *AtMAP11* was found to be strongly transiently activated in flg22- and elf18-triggered immune responses^18^. *AtMAPK15* has been shown to be involved in the regulation of pollen tube growth^45^. Here, we identified that the putative homolog of AtMAPK15, BrMAPK15, probably functions in the disease resistance response in Chinese cabbage. BrMAPK15’s function in the disease defense response was validated by an agroinfiltration-mediated transient overexpression system and VIGS technology (Figs. 5 and 6). The lncNAT of *BrMAPK15*, *MSTRG.19915*, was identified and was found to participate in the immune response by regulating the expression level of *BrMAPK15* (Figs. 5 and 6). Thus, these results may be extrapolated to infer the function and regulatory mechanism of MAPK15. To clarify the potential regulatory mechanisms of these lncRNA/protein-coding gene pairs, more evidence should be provided, such as that acquired using chromatin isolation by RNA purification and RNA immunoprecipitation^46^.

Because of the low expression levels and complex functional mechanisms of plant lncRNAs, research on their function is still limited. In particular, few studies on disease-related lncRNAs in plants have been reported. In this article, the transcriptional landscape of Chinese cabbage during *H. brassicae* invasion, including protein-coding mRNAs and lncRNAs, was studied thoroughly. We identified disease resistance-related lncRNAs during downy mildew infection and a candidate resistance-related lncRNA/protein-coding gene regulatory pair in Chinese cabbage, which will provide a basis for understanding the function of lncRNAs in response to downy mildew and a comprehensive understanding of the resistance regulatory mechanism, especially that of RNA-mediated gene regulation.

## Materials and methods

### Plant materials and growth conditions

Chinese cabbage (*B. rapa* ssp. *pekinensis*) inbred lines 12–85, T12–19 and 91–112 were used in this study. 12–85 (degree of infection (DI) = 8.2) and T12–19 (DI = 20.9) are highly resistant to downy mildew, while 91–112 (DI = 83.3) is susceptible. 12–85, T12–19, and 91–112 seedlings were geminated on wet filter paper at 25 °C in the dark for two days. The seedlings were then transplanted into soil in a climate chamber with a 16 h light/8 h dark and 23 °C/19 °C (day/night) regime. Plants at the two-leaf stage (20 days after sowing) were used for inoculation with the downy mildew pathogen.

### Downy mildew inoculation and leaf sample collection

Seedlings with two true leaves were subjected to downy mildew inoculation. The method of pathogen isolation was the same as that described by Yu et al. (2009)^24^. Approximately 2 × 10^5^ spores mL^−1^ of a conidial suspension of *H. brassicae* was sprayed onto the abaxial side of the leaves. After inoculation, the seedlings were incubated under dark conditions and high humidity (nearly 100%) during the first 24 h followed by high humidity with a 16 h light/8 h dark photoperiod for another 3–4 days for pathogen invasion. Leaf samples were collected at 0, 24, 72, and 120 h after inoculation. Three to five leaves were mixed into one composite sample, and three biological replicates were subjected to transcriptomic sequencing. All the seedlings were placed under high-humidity conditions for 4 h before inoculation to avoid transcriptomic changes caused by the high-humidity environment.

### LncRNA and mRNA deep sequencing

Isolation of lncRNAs and mRNAs and the construction of cDNA libraries were performed by the Beijing Genomics Institute (BGI, Shenzhen, China). High-quality total RNA was extracted using TRIzol (Invitrogen, Waltham, MA, USA), according to the manufacturer’s protocols. Ribosomal RNAs (rRNAs) were removed using a Ribo-Zero^TM^ Plant Kit (Illumina, San Diego, CA, USA). Strand-specific sequencing libraries were constructed according to previously described protocols^47^. First-strand cDNA was synthesized using random hexamer primers and reverse transcriptase (Invitrogen). Second-strand cDNA was synthesized using RNase H (Invitrogen) and DNA polymerase I (New England BioLabs, Ipswich, MA, USA). The sequencing libraries were subsequently generated using rRNA-free RNA with a NEBNext Ultra Directional RNA Library Prep Kit for Illumina (New England BioLabs) and sequenced using high-throughput sequencing on an Illumina HiSeq Xten paired-end 150 (PE150) instrument.

### Identification of lncRNAs and mRNAs

Clean reads were obtained by removing reads with adapters, reads with poly-N sequences and low-quality reads. The Q20 (base ratio > 20), Q30 (base ratio > 30), and GC contents of the obtained clean data were then calculated. The *B. rapa* genome sequences were downloaded from http://brassicadb.org/brad/. The clean reads of a single sample were mapped to the reference genome by TOPHAT^48^. The alignment was then inputted into StringTie, which assembled and quantified the transcripts of each sample^49^. Finally, the assembled transcripts were merged together into a uniform set of transcripts for all samples by StringTie^49^. The assembled transcripts were annotated using the Cuffcompare program of the Cufflinks package^50^. The known protein-coding transcripts were identified according to the annotations of the *B. rapa* genome sequences. The remaining unknown transcripts were used to screen for putative lncRNAs. First, transcripts <200 nt in length and with less than three reads were excluded. The coding potentials of the remaining transcripts were then calculated using the coding potential calculator (CPC) and the coding–noncoding index (CNCI)^51,52^. A transcript with a CPC value <–1 and a CNCI value lower than 0 was considered to be noncoding. Finally, the class code “u”, representing lincRNAs, the class code “x”, representing lncNAT, the class code “o”, representing the sense transcripts, the class code “I”, representing the intronic transcripts, and the class code “p”, representing the divergent lncRNAs, were assigned. The lincRNA/protein-coding gene pairs were restricted to nearby 5 kb regions and those nonoverlapping within 1 kb from protein-coding genes.

### Identification of differentially expressed lncRNAs and mRNAs

We used Cuffdiff (v2.1.1) software (Berkeley, CA, USA)^50^ to calculate the FPKM values of lncRNAs and coding mRNAs. We analyzed the differential expression of the lncRNAs and mRNAs before and after *H. brassicae* inoculation at 24, 72, and 120 hpi. For all pairwise comparisons, the expression levels of lncRNAs and mRNAs with corrected *p*-values < 0.05 and absolute fold-change values > 2.0 were considered to be differentially expressed^53^.

### Gene Ontology enrichment and cluster analysis

All the GO terms in this study were annotated using agriGO^54^ in comparison with those of the reference genome background (*P* < 0.01). The GO terms of biological processes (P) were selected for further analysis. The most significant GO terms were selected as disease response cluster annotations. MeV (v4.9) software (http://mev.tm4.org/) with the *K*-means method was used for cluster analysis. The figure of merit (FOM) was used to determine the optimal cluster number^55^.

### Prediction of putative *cis*- and *trans*-targets of lncRNAs

The transcription of lncRNAs has been implicated in the regulation of the expression of genes in close genomic proximity (*cis*-acting regulation) and in the targeting of distant genes (*trans*-acting regulation) via multiple mechanisms^6,56^. Therefore, analyses of genomic colocalization (<10 kb) of the lncRNAs and mRNAs were performed according to previously described methods^57^. In addition, the formation of near-complementary lncRNA–target duplexes is also an important way to regulate the expression of *trans*-targets^6^. The *trans*-targets of lncRNAs were predicted by the complementarity of lncRNAs and their targets with expression levels that were markedly different under Pi-deficient conditions using RIsearch^58^.

### Identification and phylogenetic analysis of *MAPK* genes

The sequences of *MAPK* genes in *B. rapa* were downloaded from the Chinese cabbage database (BRAD, http://Chinese cabbagedb.org/brad/). Moreover, sequences of *AtMAPK*s in Arabidopsis were retrieved from The Arabidopsis Information Resource (http://www.arabidopsis.org/). The full-length amino acid sequences of AtMAPKs and BrMAPKs were used for the construction of a phylogenetic tree with a neighbor-joining method by MEGA 7.0, with the following parameters: pairwise deletion mode, Poisson correction, and bootstrapping (1000 replicates)^59^.

### Gene and lncRNA cloning and construction

The reference sequences of *Bra003834* (*BrMAPK15*) and *MSTRG.19915* were derived from the Brassica database (http://brassicadb.org) and RNA-seq data, respectively. The primers used for cloning were designed based on the reference sequences. *BrMAPK15* and *MSTRG.19915* were amplified from the samples from 91–112–0 and 12–85–120 h, respectively. The PCR products were then ligated into the pCR8/GW/Topo^TM^ entry vector (Invitrogen) and sequenced. Sequence similarities were calculated using DNAMAN version 6 (Lynnon Biosoft, San Ramon, CA, USA). The sequences of *BrMAPK15* and *MSTRG.19915* were cloned into a pCAMBIA2300 vector between the KpnI and XbaI restriction sites using a seamless cloning kit (Biomed, Lot. CL117, Beijing, China). These recombinant plasmids were then transformed into *Agrobacterium tumefaciens* strain GV3101 for Agrobacterium-mediated transient overexpression. Specific 40 nt oligonucleotides were identified in the sequences of *BrMAPK15* and *MSTRG.19915*. The palindromic 80-nt oligonucleotides corresponding to a dimer of these 40-nt oligonucleotides were then inserted in a pTY-S vector at the SnaBI restriction site^60^. The resulting constructs were used in VIGS experiments. The sequences of all the primers used in this study are listed in Supplementary Table S10.

### VIGS

The VIGS method was performed as described by Muntha et al. 2019^61^, with some modifications. Two recombinant pTY-S plasmids (the empty pTY-S plasmid was given by Professor Yang Jinghua) containing specific palindromic 80-nt oligonucleotides of *BrMAPK15* and *MSTRG.19915*, respectively, were extracted in large quantities for virus infiltration. Two-week-old seedlings grown at 20 °C/22 °C under a 16 h/8 h light/dark cycle were applied for virus infiltration. First, silicon carbide powder was sprinkled onto the surface of a fully expanded leaf. Gentle friction was then applied on the leaf using fingers to help make tiny wounds, which facilitated the entrance of the exogenous DNA. Afterward, 8 μL of a solution of purified pTY-S recombinant plasmid (300 ng/μL) was applied evenly onto the leaf. The pTY-S recombinant plasmid was infiltrated into leaves for 2–5 min, after which they were washed with water. Two to four leaves per seedling were used for infiltration. The infiltrated seedlings were placed under dark conditions for 16 h. Three or four infiltrations were performed over 2 weeks. At 2 weeks post-infiltration, the seedlings were inoculated with *H. brassicae* as described above. Five days after inoculation, the phenotypes were observed, and the gene expression levels were measured. Silencing of the *PDS* gene causes photobleaching. Therefore, the pTY-PDS vector was used as a positive control.

### Agroinfiltration

The method of transient expression mediated by agroinfiltration was performed as described by Zhang et al.^43^. The cotyledons of 7-day-old 91–112 and 12–85 seedlings were used in the agroinfiltration experiment. Cotyledons without infiltration were used as controls to measure gene expression. Cotyledons infiltrated with empty vectors were used as controls for the phenotype investigation. At 3 days after infiltration, 3–5 infiltrated cotyledons from each sample were mixed together for qRT-PCR analysis. The RNA samples in three biological replicates were then subjected to qRT-PCR analysis. After 3 days, the infiltrated cotyledons were inoculated with *H. brassicae*. The phenotype of the infiltrated cotyledons was investigated at 5 days after inoculation. An infected cotyledon, of which more than half the leaf area had observable sporulation, was considered to be a susceptible cotyledon. The expression levels of infiltrated genes and the proportions of susceptible cotyledons were derived from three independent experiments.

### RNA extraction and qRT-PCR

Total RNA was extracted from leaves using a plant RNAprep Pure Kit (Lot. DP441, TIANGEN, Beijing, China). A PrimeScript™ RT Reagent Kit (Takara, Osaka, Japan) was used to reverse transcribe RNA into cDNA. Real-time PCR was then performed using SYBR Green I Master Mix (Roche, Basel, Switzerland) with cDNA as a template and quantified using a Light Cycler 480 II instrument (Roche). The reaction conditions were as follows: initial denaturation at 95 °C for 3 min, followed by 40 cycles of 95 °C for 15 s, 60 °C for 30 s, and 72 °C for 45 s. PCR amplification was followed by heating for 1 min at 60–95 °C for melting curve analysis. Each sample reaction was performed for three replicates using 5 μL of Master Mix, each primer at 0.25 μM, 1 μL of diluted cDNA, and DNase-free water in a final volume of 10 mL. The PCR products were sequenced to confirm the gene-specific amplification.

### Data access

The raw sequence data generated from the Chinese cabbage inbred lines 91–112, T12–19 and 12–85 in this paper have been deposited in the Genome Sequence Archive (GSA)^62^ in the National Genomics Data Center^63^, Beijing Institute of Genomics (China National Center for Bioinformation), Chinese Academy of Sciences, under accession number CRA003552, which is publicly accessible at https://bigd.big.ac.cn/gsa.

## Supplementary information

Revised-supplementary material

Revised-supplemental tables
