# Supplementary material for: Identification of long noncoding RNAs involved in resistance to downy mildew in Chinese cabbage
Source: Hortic Res. 2021 Mar 1;8:44. doi: 10.1038/s41438-021-00479-1 (PMC7917106; doi:10.1038/s41438-021-00479-1)
Supplement: Supplementary file 1 — Revised-supplementary material [file 41438_2021_479_MOESM1_ESM.docx]

Supplementary Material

Identification of long noncoding RNA involved in resistance to downy mildew in Chinese cabbage

Bin Zhang, Tongbing Su, Peirong Li, Xiaoyun Xin, Yunyun Cao, Weihong Wang, Xiuyun Zhao, Deshuang Zhang, Yangjun Yu, Dayong Li, Shuancang Yu*, Fenglan Zhang*

*** Correspondence:** Shuancang Yu, yushuancang@nercv.org, Fenglan Zhang, zhangfenglan@nercv.org

# Supplementary Figures and Tables

## Supplementary Figures


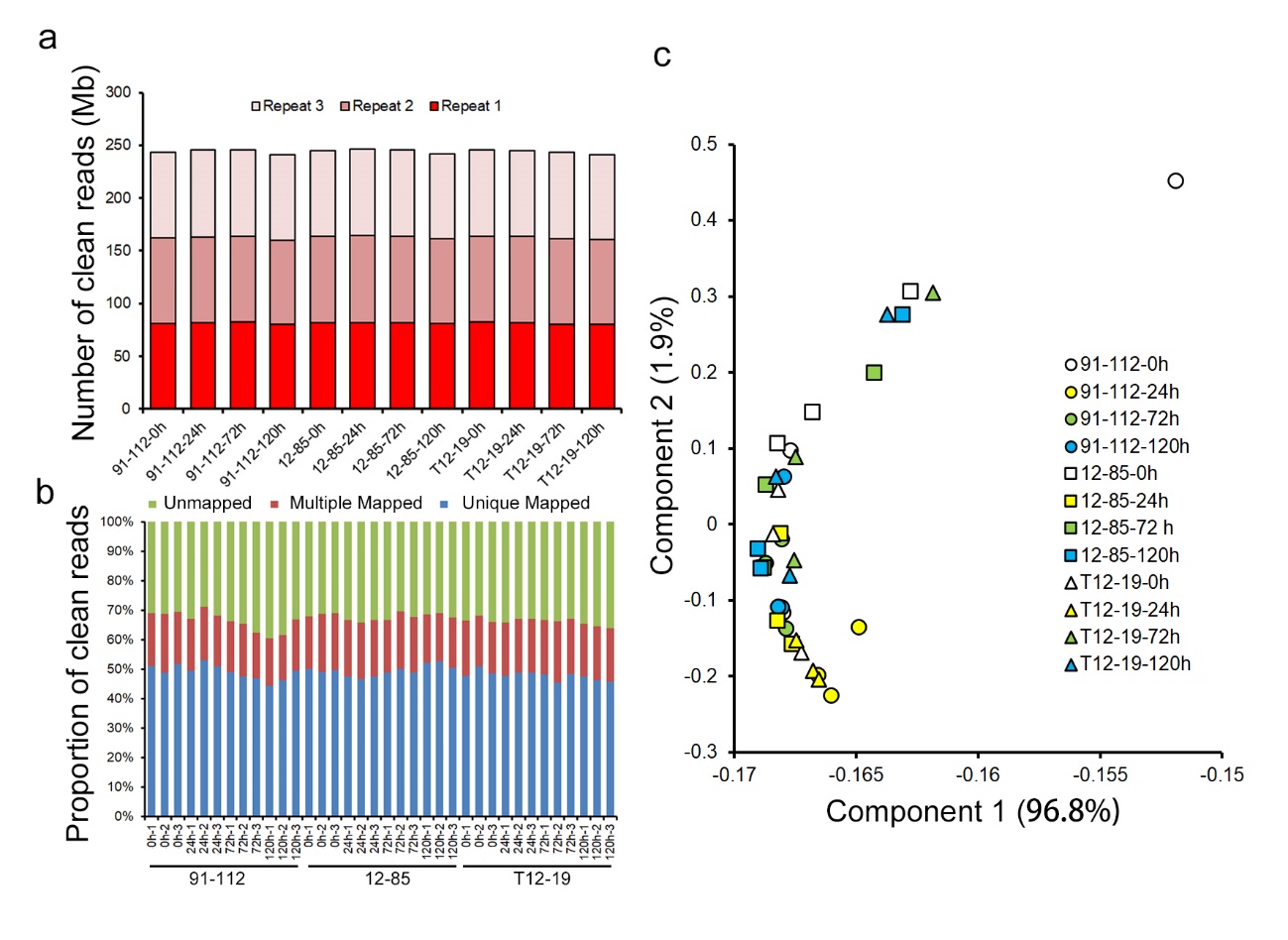


**Supplementary Figure 1. The transcriptome sequencing of 36 samples (including three biological repeats).**

a. The numbers of clean reads; b. The proportions of clean reads that were unmapped, multiply mapped, and uniquely mapped to the genome; c. The PCA analysis of these 36 samples.


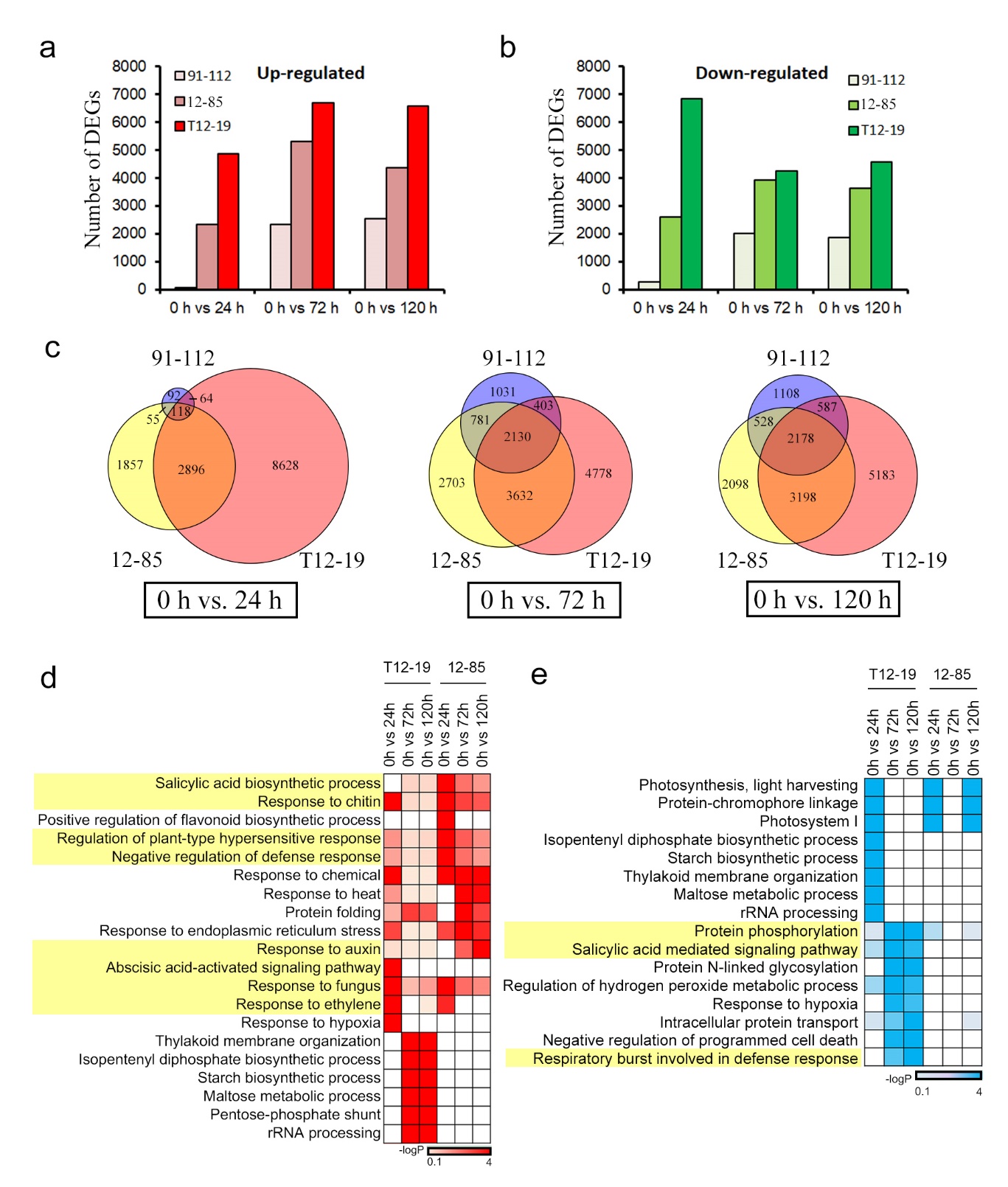


**Supplementary Figure 2. Figure S2. Identiﬁcation and characterization of DEGs in 91-112, 12-85, and T12-19.**

a. The numbers of upregulated DEGs at 24, 72, and 120 hpi compared with that at 0 hpi; b. The numbers of downregulated DEGs at 24, 72, and 120 hpi compared with that at 0 hpi; c. Venn diagram analysis of DEGs in 91-112, 12-85, and T12-19 at each time point after inoculation; d. GO analysis of 12-85- and T12-19-specific upregulated DEGs; e. The GO analysis of 12-85- and T12-19-specific downregulated DEGs.


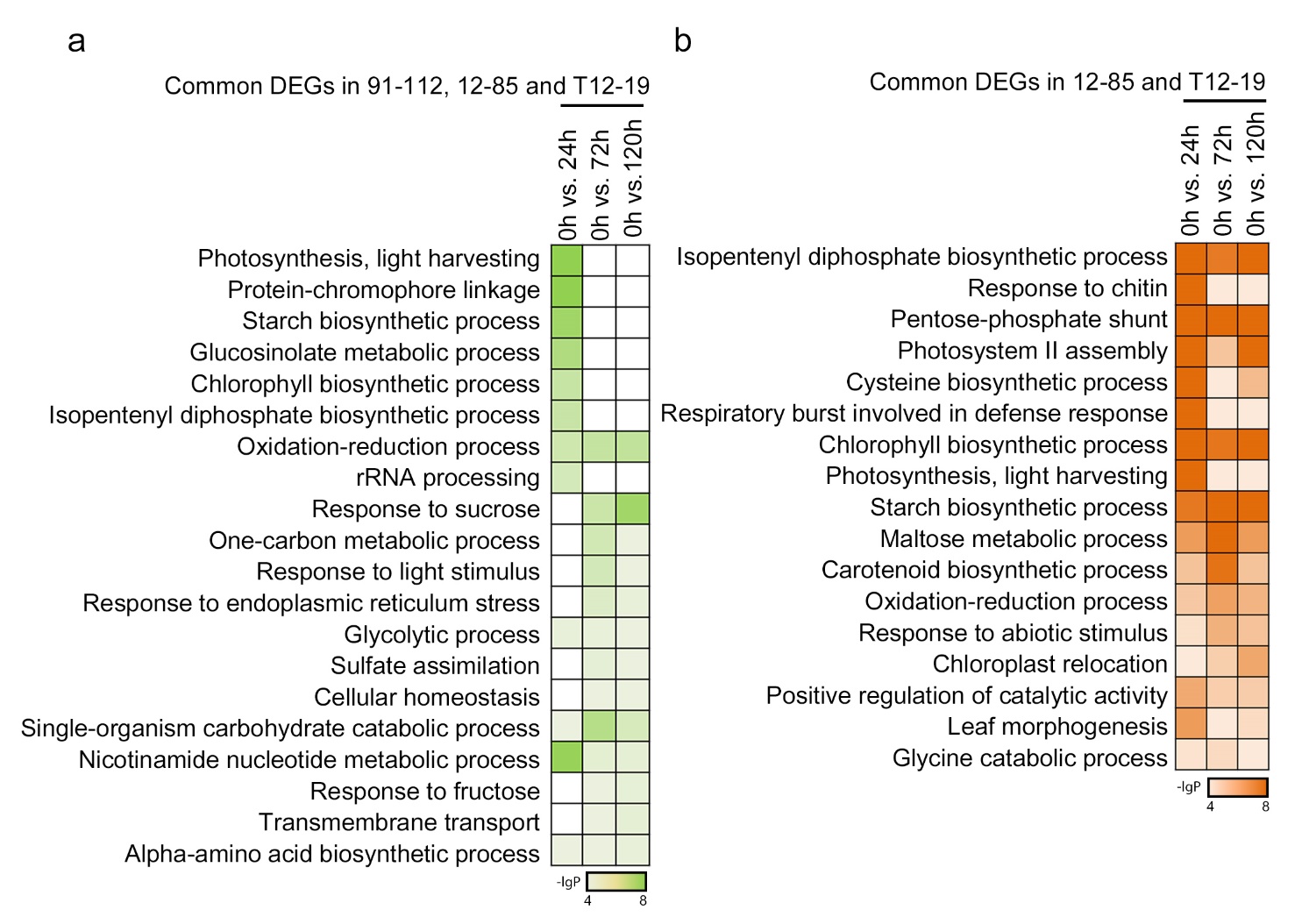


**Supplementary Figure 3. GO analysis of DEGs in 12-85 and T12-19.**

a. GO analysis of upregulated DEGs in 12-85 and T12-19; b. The GO analysis of downregulated DEGs in 12-85 and T12-19.


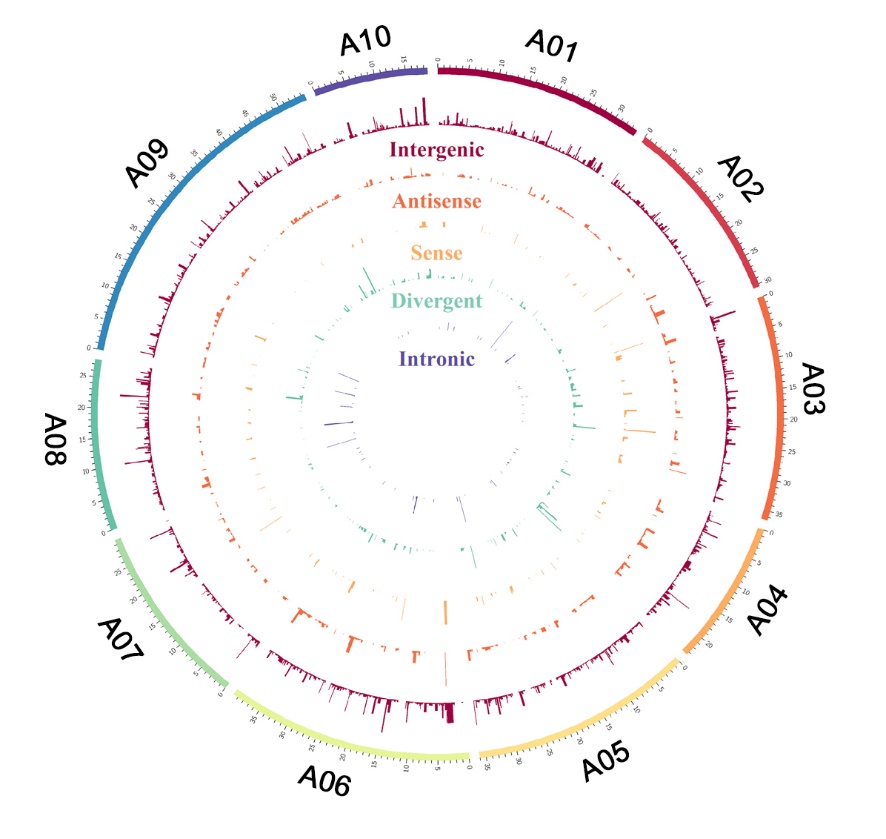


**Supplementary Figure 4. The expression levels and genome locations of different kinds of lncRNAs.**


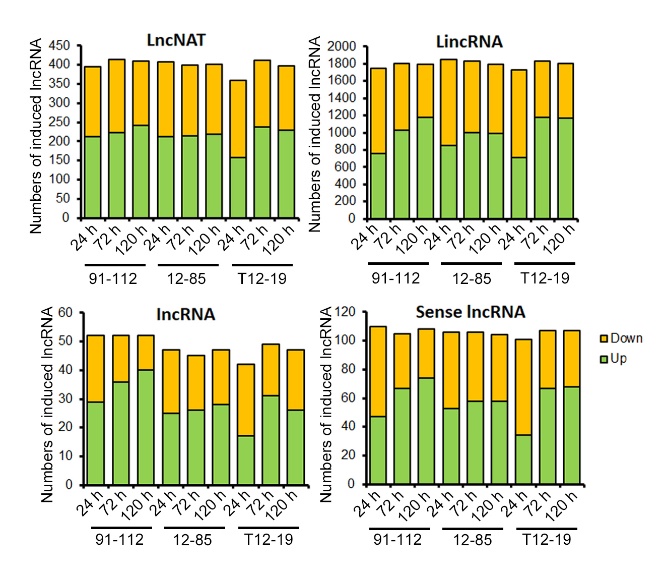


**Supplementary Figure 5. The numbers of up- and downregulated lncRNAs**

(a), (b), (c) and (d) are *BrLRR1*, *BrLRR2*, *BrRLP47* and *BrRLP48*, respectively. The upper, middle and lower sequences aligned are from reference, BY and MM genomes, respectively.


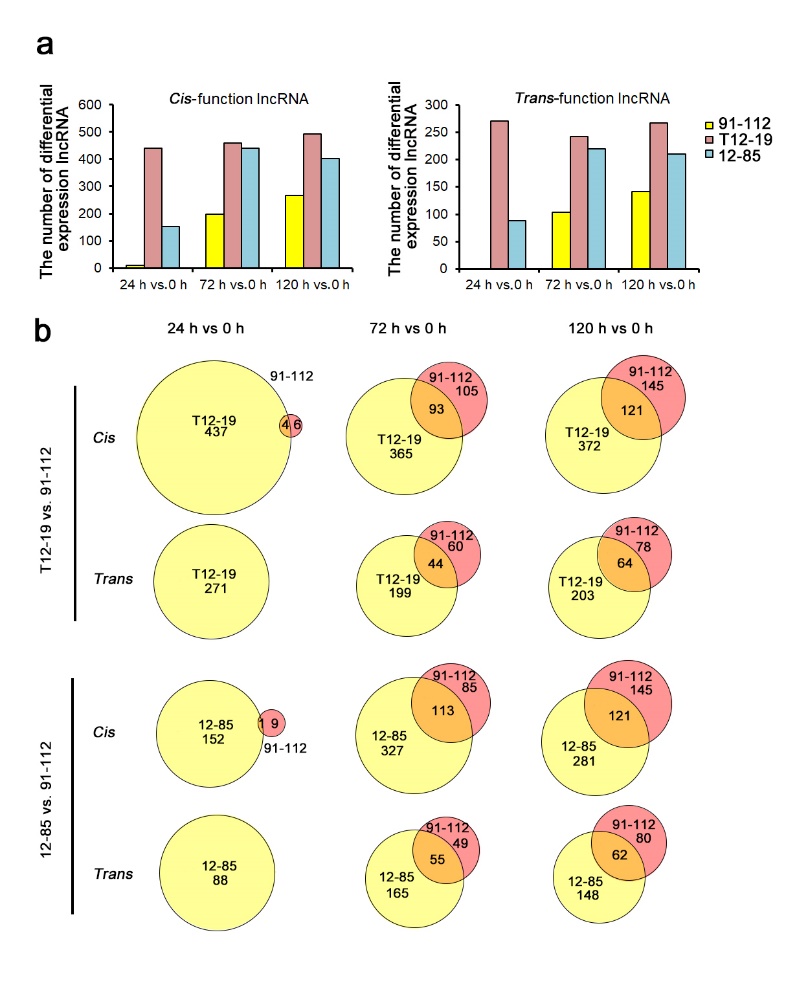


**Supplementary Figure 6. Identiﬁcation and characterization of cis- and trans-functional lncRNAs.**

a. The numbers of cis- and trans-functional lncRNAs at each time point in 91-112, 12-85, and T12-19; b. Venn analysis of cis- and trans-functional lncRNAs at each time point in 91-112, 12-85, and T12-19.


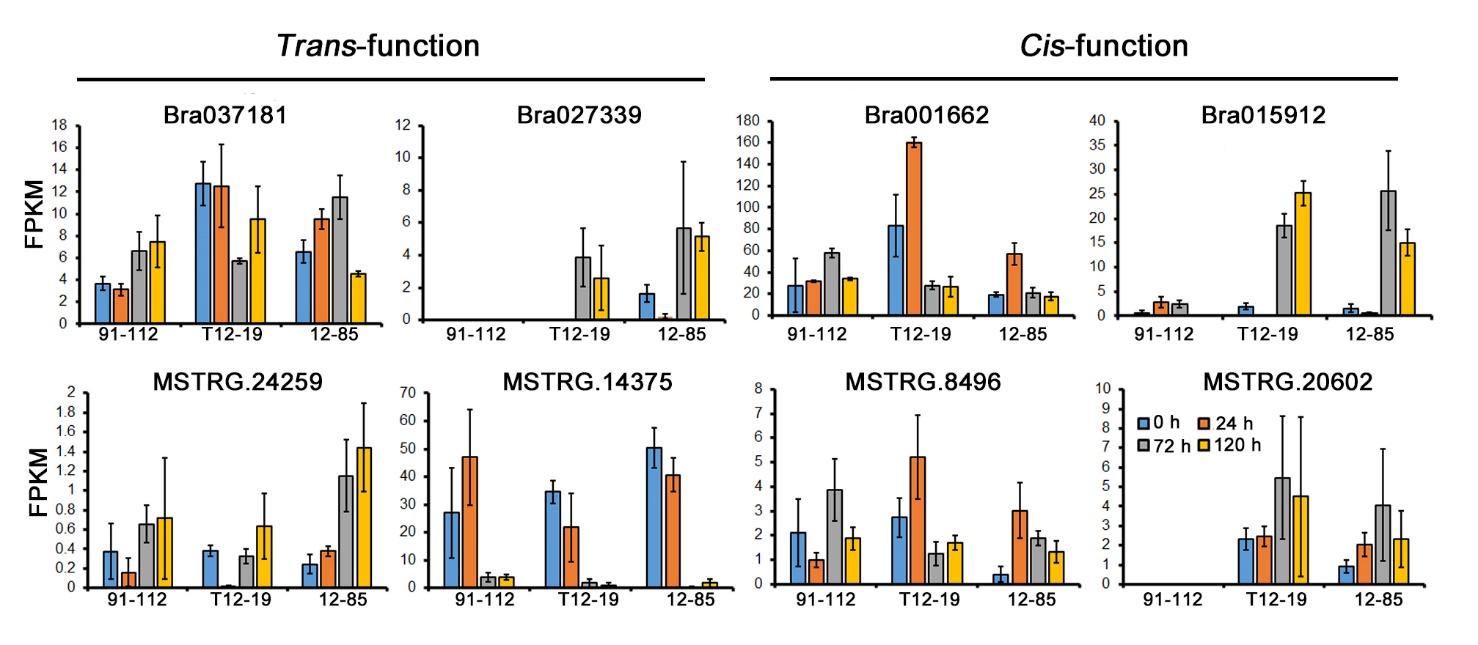


**Figure S7. The expressions of trans- and cis-functional lncRNA/protein-coding gene pairs in RNAseq data.**

The four panels on the left are the FPKM values of two trans-function lncRNAs/protein-coding gene pairs in all samples. The other ones are the cis-function lncRNAs/protein-coding gene pairs.


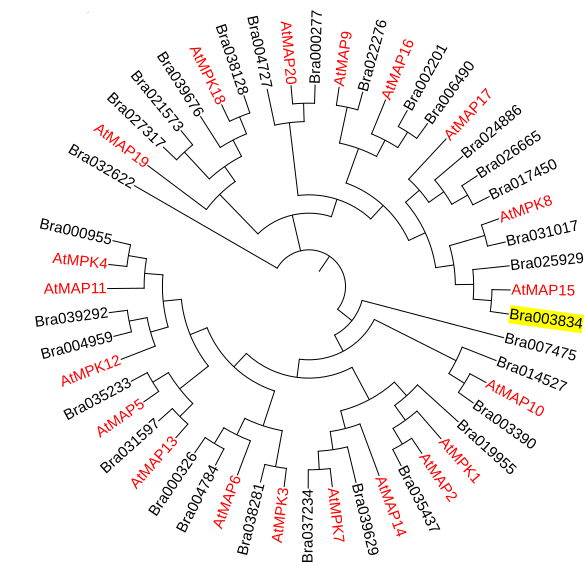


**Figure S8. Phylogenetic analysis of MAPK family proteins in Arabidopsis and *Brassica rapa*.**

The MAPK proteins from Arabidopsis are in red. BrMAPK15, which was identified in this study to function in downy mildew resistance response, is highlighted by yellow.

1.1 Supplementary Tables

The Supplementary Tables for this article can be found in the attached file with excel format.
